# Lessons learned and policy implications from an organizational reform of the World Health Organization African region country offices: a policy brief

**DOI:** 10.3389/fpubh.2025.1558268

**Published:** 2025-08-05

**Authors:** Abdulmumini Usman, Joseph Cabore, Olushayo Oluseun Olu, Ndoungou Salla Ba, Patrick Kabore, Joseph Okeibunor, Alex Gasasira, Matshidiso Moeti

**Affiliations:** World Health Organization African Regional Office, Brazzaville, Republic of Congo

**Keywords:** transformation agenda, functional review, organizational reforms, World Health Organization African region, World Health Organization country offices, Africa

## Abstract

A decade ago, the World Health Organization (WHO) faced global calls for significant reforms to shift its focus from donor-driven priorities to those determined by its Member States. This demand was driven by political and financial pressures aimed at enhancing effectiveness and efficiency in service delivery. The WHO Regional Office for Africa was particularly challenged, and the 2014–2015 Ebola outbreak further amplified the need for transformative changes. In response, the World Health Organization Regional Director launched the Transformation Agenda in 2015, aiming to improve the organization’s efficiency and responsiveness. Within this reform agenda, a major restructuring of the 47 World Health Organization Country Offices, known as the Functional Review, was conducted between 2017 and 2019. This article reflects on the experiences and lessons from the Functional Review process, providing insights, policy options, and recommendations for future reforms. Key lessons from the Functional Review include the need for sufficient resources, stakeholder engagement, flexibility, and clear communication during organizational reforms.

## Introduction

A decade ago, there was a global call for the World Health Organization (WHO) to undertake significant reforms. At the heart of this demand was the need to shift the organization’s focus from donor-driven priorities to those determined by its Member States (MSs) (1). Such calls for organizational reform are often driven by political and financial pressures aimed at enhancing effectiveness and efficiency in service delivery (2). Much of the call for reforms was directed at WHO’s Regional Offices, particularly the WHO Regional Office for Africa (WHO/AFRO), which was widely regarded as the most challenged. The incoming Regional Director at the time faced strong appeals to initiate transformative changes (3). This call for reform was further amplified by the 2014–2015 Ebola outbreak in West Africa, which exposed critical weaknesses in WHO’s emergency response capacity while simultaneously underscoring the organization’s vital role in supporting its MSs to address public health threats, especially those related to global health security (4, 5).

This push for reform is not unique to WHO. Similar demands have long been directed at the broader United Nations (UN) system, calling for changes to both its political architecture and operational modalities. However, consensus on the scope and direction of such reforms has remained elusive (6). Since 1974, UN MSs have engaged in discussions on institutional reform, culminating in the adoption of restructuring principles by the General Assembly in 1978 (7). Nevertheless, calls for further reform have persisted, with debates continuing into 2024 (8).

Reform efforts in the health sector have been a global phenomenon, occurring across countries regardless of their level of development. These efforts have typically involved changes in institutions and service delivery systems (9). Many national health sector reforms have focused on key areas, such as policy, financing, governance, and service organization (10, 11). Despite these global trends, skepticism has persisted among observers regarding WHO’s willingness and capacity to implement meaningful institutional change (12). Some critics have been of the view that WHO regional leaders were more interested in regional politics than reform efforts (13). Others have noted that the considerable autonomy exercised by WHO regional offices continues to hinder the organization’s ability to effectively implement coherent global health policies and reform itself (14).

In response to the widespread calls for organizational reforms, the incoming Regional Director of WHO/AFRO launched a comprehensive reform initiative in 2015, known as the Transformation Agenda (TA). This initiative was designed as both a vision and a strategy for change, with the overarching goal of shaping the organization into “the WHO that staff and stakeholders want” (15). A central component of the TA was the development of the Africa Health Transformation Program, 2015–2020, a strategic vision for achieving Universal Health Coverage (UHC). This program aimed to enhance WHO/AFRO’s capacity and reposition its work to be more effective, efficient, and results-oriented (16). The primary objective was to deliver more impactful technical and policy support to its MSs by aligning staff competencies with the needs of MSs and ensuring that resource allocation was responsive to national health priorities.

As part of the TA, a major restructuring of the 47 WHO Country Offices (COs) in the Region was carried out between 2017 and 2019. This restructuring, known as the Functional Review (FR), was a human resource and organizational alignment exercise. It aimed to ensure that the workforce and operational structures were better matched to the health situations, needs, and strategic priorities of the organization’s MSs.

This article reflects on the experiences and lessons derived from the FR process. It provides insights, explores relevant policy options, and proposes key recommendations that may be of value to other multilateral organizations embarking on similar institutional reform initiatives. In light of continued global calls for health sector and organizational reforms, the findings presented here contribute to a broader framework for guiding future reform efforts.

## Organizational context of the functional review exercise

The WHO serves as the UN’s specialized agency for health, encompassing six regions, including the WHO African Region (WHO/AFR). This region is responsible for coordinating public health services and providing technical assistance to the 47 WHO African MSs. The WHO/AFR is governed by a Regional Committee of Health Ministers. The WHO/AFR Secretariat is organized into the African Regional Office, known as the WHO/AFRO, based in Brazzaville, Congo, and 47 COs predominantly located in sub-Saharan Africa. The Secretariat is led by a Regional Director, who is supported by country representatives overseeing the organization’s activities at the national level.

The WHO/AFR COs execute the organization’s core functions at the country level, which include providing technical assistance to MSs for the adaptation and implementation of international and regional health norms and standards, articulating evidence-based policy options, shaping the health research agenda, and supporting the strengthening of national governance and leadership. In large and emergency-affected countries, the COs have sub-national offices referred to as sub-offices. The technical, administrative, and financial aspects of the organization’s work at the country level are led by the country’s representatives.

## The functional review process

The FR process commenced with a comprehensive analysis of national policy documents and WHO operational reports to gain a contextual understanding of each country prior to in-country visits. This was followed by in-country visits during which extensive stakeholder consultations were conducted. These consultations aimed to identify and validate the priorities and expectations of various actors regarding WHO’s role and performance and included a structured partner survey and in-person discussions.

Subsequently, a rigorous tabletop exercise was undertaken to synthesize findings from the consultations and to translate stakeholder expectations into specific functional roles, human resource requirements, and proposed structural adjustments for COs. The financial feasibility of the proposed structures was assessed using the WHO standard salary cost, also referred to as the post cost average, to estimate the cost implications of implementation.

Once the revised CO structures and specific recommendations were finalized, they were presented to WHO staff, MSs, and external partners, including donors, through established platforms such as staff meetings, donor roundtables, and individual briefings. To ensure consistency and accountability in the implementation phase, a comprehensive set of guidelines was developed. These guidelines, monitored by a high-level oversight committee within the WHO/AFRO, provided detailed instructions on managing the human resource implications of the FR. They served as the operational framework for translating the FR outcomes into action in a harmonized and transparent manner across the WHO/AFR.

## Key observations and lessons learned from the functional review and their policy implications

Several lessons with policy implications that would be useful to other national and international organizations seeking to reform themselves were learnt in the process of implementing the FR.

First, organizational reform exercises are resource- and time-intensive; thus, there is a need to avail sufficient resources to carry out the various aspects of the exercise. The initial plan to complete the FR in 2 years was extended to 4 years due to the need to re-strategize sometimes. Additionally, the WHO needed to recruit long-term FR staffing as opposed to the initial consultancy arrangement. These increased the FR cost beyond the original estimates. Thus, it is critical to plan and allocate the required budget elements, such as salaries, allowances, travel and meeting costs, and funds to implement the findings before embarking on major reforms. Second, the endorsement of organizational reforms by stakeholders may not translate into funding for their implementation. Despite positive engagement and support for the FR outcomes by stakeholders, especially donors, very few were willing to make flexible investments toward their implementation. Thus, lack of funding became a major constraint to the implementation of the outcomes of the FR, leading to major delays, necessitating a change in approach.

Third, the coronavirus disease 2019 (COVID-19) pandemic, which began in 2019 and spread to Africa in 2020, was not anticipated and diverted attention from the implementation of the FR. Thus, adequate provision should be made for such unforeseen circumstances when planning organizational reforms. Fourth, flexibility is essential when implementing the outcomes of organizational reforms. In this case, flexibility in the staffing model for the new country office structures permitted the introduction of novel ways of working to address the gaps in staffing needs. These included prioritizing functions based on available funds, assigning staff to work across multiple countries, utilizing temporary surge support during periods of increased workload, and collaborating through partners.

Fifth, major organizational reforms such as the FR involve making difficult decisions that could affect staff careers and livelihoods. Such decisions often result in disappointment, stress, and anxiety among staff. Despite regular communication from the Regional Director aimed at reducing tension, not all staff were reassured—and rightly so, as some staff members ultimately lost their jobs. Several staff members who initially supported the FR reacted very negatively when their positions were discontinued in the new structure, putting them at risk of being laid off. However, the need to minimize staff anxiety should be carefully weighed against creating false hopes that may later result in legal issues. Additionally, another dilemma was striking a balance between the proportion of secured, predictable funds that should be used to fund new high-priority functions and sustaining existing and essential but less prioritized functions.

## Discussion and recommendations

Reforming organizations through restructuring, defined as the act of changing the business model of an organization to transform it for the better (7), is not a novel initiative. The UN itself has undergone restructuring as part of its reforms aimed at improving efficiency (17), resulting in the reorganization of existing structures and creation of new ones as the case may be in 2019, including the Development Coordination Office, the Department of Political and Peacebuilding Affairs, and the Department of Management Strategy, Policy and Compliance (18). These reforms are often pushed and funded by international funding agencies such as the International Monetary Fund (19). The world is constantly changing as a result of globalization and the emergence of more global actors, especially in health and international cooperation. This is challenging the authority of international organizations such as the WHO, even in its traditional role of normative guidance and measuring global health trends (20). Thus, there will be a continuous need for the restructuring of organizations in response to these changes in the operational space. The findings of the FR process, including the lessons learned, will therefore contribute to such efforts.

Stakeholder consultations are important in the conduct of a restructuring exercise, as they provide opportunities to obtain feedback on the organization. All stakeholders welcomed the process of the FR; however, they had different perceptions of WHO and may not be fully aware of its functions. This occured despite the existence of a communication framework since 2017, which recognizes communication as integral to WHO’s work (21). In addition, stakeholder engagement provides an opportunity to communicate important messages to stakeholders. For example, only approximately 15% of the stakeholders consulted were aware of WHO’s normative function. This lack of precise knowledge about the organization’s functions may contribute to negative impressions of its performance and overall image. This masking of its normative functions at the country level may be linked to the often-cited focus on donor-driven, vertical, project-based implementation at the expense of evidence generation and standard setting (22).

There is an ongoing debate on the role that the WHO should play in the current crowded global health landscape, with some suggesting that the organization should focus on health governance and technical assistance, ceding operational functions to other organizations (23). Others argue that narrowing WHO activities could hamper its leadership in global health governance (24). Observations during the FR suggest that the normative functions of the WHO should not be excluded from its operational functions. However, decisions about which functions the organization should perform should be context-specific and clearly communicated to all stakeholders, especially at the country level.

Meeting stakeholder expectations regarding the type of support they require, as revealed by the FR, will necessitate staffing with competencies that differ from those that currently exist. Such staffing structures should consider the mix of skills (occupational types, grade levels, and numbers of positions in each occupational category) required to achieve the strategic objectives, functional interrelationships, and efficient work processes in providing technical assistance to MSs. Staffing these structures requires the engagement of new personnel with both local and international experiences and a significant overall increase in staff numbers in most COs, as reported earlier (25).

Another expectation of WHO is the need to transform from aligning to donor priorities to addressing priorities of MSs (1). However, the actions of the MSs and the donors regarding the funding of the WHO often conflict with this expectation. In many instances, several bilateral donors who are themselves MSs often insist on their priorities over those of the receiving MS. This lack of flexibility to realign MSs’ contributions to what they expected the organization to do has been a consistent challenge, even though WHO has recorded improvements in financing reform, predictability, and transparency (26). The call for restructuring organizations should therefore be accompanied by a commitment to providing flexible funds to implement the outcomes of such restructuring exercises.

## Conclusion

This article highlights the importance of establishing clear principles at the outset of major organizational reforms, particularly within complex global health organizations such as the WHO. Setting such ground rules and preparing for unforeseen circumstances ensures coherence and alignment throughout the reform process, especially when engaging a wide array of stakeholders with differing perspectives and interests. In cases where multiple organizational components are involved, as in the case with the FR, identifying areas of convergence is essential for defining overarching priorities that can be addressed. The distinct needs and contexts of individual countries can then be addressed during the implementation phase, ensuring both relevance and adaptability. However, reform processes are often confronted with challenges such as resistance to change, long-term organizational liabilities, and a shortage of resources. Anticipating these risks and developing mitigation strategies early is vital for effective and sustainable organizational reforms. Moving forward, the WHO/AFRO and its COs should prioritize the full and sustained implementation of the FR.

## Recommendations

Enhancing capacity and performance at the country level, particularly in areas such as evidence-based health policy dialogue and guidance, health coordination, technical brokerage, and delivery of high-level expertise, will be crucial. These improvements are essential for ensuring the WHO’s continued relevance and effectiveness within an increasingly complex and competitive global health environment. The organization’s Fourteenth General Program of Work (GPW 14), with its strong emphasis on country-level impact, represents a promising step in this direction and aligns closely with the strategic objectives and insights emerging from the FR.
